# Impact of reduced pancreatin and bile on Fe and Zn bioaccessibility assessment using the INFOGEST *in vitro* digestion method with stable isotopic labelling^☆^

**DOI:** 10.1016/j.fochx.2026.103483

**Published:** 2026-01-02

**Authors:** Alexandre Minami Fioroto, Molly Muleya, Lolita Wilson, Kaja Kristensen, Ruth Price, David A. Gray, Eduardo Purgatto, Elizabeth H. Bailey

**Affiliations:** a Food Research Center (FoRC), University of São Paulo (USP), São Paulo, SP, Brazil; b Department of Food and Experimental Nutrition, Faculty of Pharmaceutical Sciences, University of São Paulo (USP), São Paulo, SP, Brazil; c School of Biosciences, Division of Food, Nutrition and Dietetics, University of Nottingham, Sutton Bonington Campus, Loughborough, Leicestershire LE12 5RD, UK; d School of Biosciences, Division of Agriculture and Environmental Sciences, University of Nottingham, Sutton Bonington Campus, Loughborough, Leicestershire LE12 5RD, UK

**Keywords:** Minerals, Pancreatin, Bile, *In vitro* digestion, Bioaccessibility, Digestibility, Isotopic labelling

## Abstract

In a previously proposed stable isotope approach based on the INFOGEST protocol, pancreatin and bile amounts were reduced due to their high mineral content, which interferes with Fe and Zn measurements. The present study examined the impact of reagent reduction on macronutrient digestibility and, consequently, mineral release in ten food samples. Macronutrient digestibility was higher under INFOGEST conditions. However, Fe and Zn solubility were not consistently modified across matrices, although, a general trend of increased solubility was observed in the standard INFOGEST method. Fe solubility increased by 64–91 % in cereals, beans, and nuts but decreased by 48 % in sweet potato. Similarly, Zn solubility increased by 11–75 % in oats, nuts, and leafy vegetables, while it decreased by 18–64 % in potatoes and beans. Overall, the influence of enzymatic action and background minerals could not be isolated, thus standardisation of the INFOGEST method for mineral bioaccessibility assessment is urgently needed.

## Highlights

• High mineral background in fluids interferes with Fe and Zn bioaccessibility measurements.
• Reducing the amounts of pancreatin and bile in the fluids can impair protein and starch hydrolysis.
• Fe and Zn solubility varied across food matrices and *in vitro* digestion methods.
• Fe and Zn solubility tends to be higher under standard INFOGEST conditions.

## 1 Introduction

*In vitro* digestion methods are widely employed to assess the bioaccessibility of minerals and other nutrients. These methods simulate physiological conditions such as enzymatic activity, pH, electrolyte concentrations, temperature, and digestion time (Hur, Lim, Decker, & McClements, 2011; Minekus et al., 2014; Thakur et al., 2020). Although *in vitro* models cannot fully replicate all physiological processes, they serve as effective alternatives to *in vivo* methods and generally show a good correlation with *in vivo* results (Aragón, Ortiz, & Pachón, 2012). Moreover, *in vitro* methods are simpler, faster, less expensive, and free from ethical concerns (Hur et al., 2011; Minekus et al., 2014; Thakur et al., 2020).

Several *in vitro* digestion models have been described in the literature; however these methods often exhibit significant variations in digestion conditions *i.e.*, digestion phases simulated (*e.g.* oral, gastric, and intestinal), composition of the digestion fluids, types and activity of digestive enzymes and pH conditions (Hur et al., 2011; Minekus et al., 2014; Sulaiman, Givens, & Anitha, 2021). Consequently, results obtained from different studies may not be directly comparable (Minekus et al., 2014).

The INFOGEST static *in vitro* digestion protocol was developed in response to the need for a standardized method. It was the result of an international network of specialists from diverse areas, including food science, nutrition, gastroenterology, and enzymology (Minekus et al., 2014). Five years after the first version of the protocol was established, some parameters were reviewed and an updated INFOGEST 2.0 method was published (Brodkorb et al., 2019).

The digestive fluids described in the INFOGEST 2.0 protocol are composed of electrolytes, enzymes (salivary α-amylase, porcine pepsin and pancreatin), and bovine bile salts (Brodkorb et al., 2019; Minekus et al., 2014). These fluids may contain minerals due to their presence in the reagents, which compromises the quantification of the bioaccessible mineral fractions. This is because during *in vitro* digestion, reagent and sample minerals enter a common pool to which they are both subjected to interactions modifying their bioaccessibility. The mineral content in the fluids depends on both the concentrations present in the electrolytic reagents and enzymes/bile and the amounts used for each phase during the *in vitro* digestion (Muleya, Young, & Bailey, 2021).

In order to distinguish the mineral concentrations derived from samples and those introduced by reagents, Muleya et al. (2021) proposed a stable isotope approach based on a modified INFOGEST static *in vitro* digestion method to assess the Fe and Zn bioaccessibility in assorted cereal and legume samples (*e.g.*, common bean, pearl millet, finger millet, maize, cowpea, velvet bean, and wheat). The reagents used in the simulated digestion fluids were isotopically labelled by the addition of ^57^Fe and ^70^Zn isotopes. The measurements of native (^56^Fe and ^66^Zn) and added isotopes (^57^Fe and ^70^Zn) allowed for the determination of the reagent's contribution to total Fe and Zn concentrations and enabled an accurate assessment of their bioaccessibility.

The contributions of pancreatin and bile to Fe and Zn levels in the digesta were much greater than those of other reagents, potentially representing over 90 % of total reagent Fe and Zn. In most cases, the Fe and Zn derived from reagents was higher than their concentrations in the samples. Moreover, saturated solutions of pancreatin and bile may introduce additional mineral binders/ligands in the system that could adsorb and precipitate Fe and Zn during digestion, affecting the bioaccessibility measurements. For that reason, the pancreatin and bile amounts were reduced (Muleya et al., 2021).

Pancreatin is a mixture of enzymes, composed of proteolytic enzymes (trypsin, chymotrypsin), α-amylase, lipase. The INFOGEST protocol standardizes the amount used during the intestinal phase based on its trypsin activity (Brodkorb et al., 2019). In order to reduce the contribution of reagent Fe and Zn, as well as their interference in mineral binding, Muleya et al. (2021) used overall proteolytic activity instead of trypsin activity as the reference, resulting in a more than 60-fold reduction in the amount of pancreatin applied. Additionally, the concentration of bile salts was reduced from 10 mM to 2 mM. This reduction was according to typical pancreatin and bile amounts used in an *in vitro* digestion method commonly used for assessment of Fe uptake by caco-2-cells (Glahn, Lee, Yeung, Goldman, & Miller, 1998).

Pancreatic enzymes play the most important role in macronutrient digestion (Layer & Keller, 2008). In addition, bile salts are essential for lipid emulsification, which enables enzymatic access, allowing not only lipid hydrolysis but also enhancing protein hydrolysis during the intestinal phase. (Asensio-Grau, Calvo-Lerma, Heredia, & Andrés, 2019; Dulko et al., 2021). Reductions in enzymes and bile concentrations during *in vitro* digestion have been reported to affect the mineral solubility, leading to variations in bioaccessibility results (Laparra, Barberá, & Farré, 2005).

The food matrix also plays a crucial role in determining the extent and kinetics of nutrient release (Capuano & Pellegrini, 2019). Mineral nutrients may be heterogeneously distributed within the food matrix and associated with different macronutrients (Shen et al., 2023). In addition, they can be physically entrapped within structural components, depending on particle size, cell integrity, or intracellular compartmentalization (Capuano & Pellegrini, 2019; Holland, Ryden, Edwards, & Grundy, 2020). Thus, insufficient pancreatin and bile amounts may reduce macronutrient digestibility and, consequently, the release of minerals associated with macromolecular structures.

Moreover, the hydrolysis products of macronutrients strongly influence mineral bioaccessibility. These compounds can chelate minerals, preventing their precipitation in the intestinal phase (Zhang, Stockmann, Ng, & Ajlouni, 2022). In addition, antinutrients released from the food matrix during digestion may bind to minerals and form insoluble complexes that reduce their bioaccessibility (Zhang, Stockmann, Ng, & Ajlouni, 2021).

Therefore, in the present study, the feasibility of employing processing methods to remove minerals from pancreatin and bile was evaluated, aiming to reduce the mineral content in the fluid while maintaining conditions as close as possible to those of the INFOGEST 2.0 protocol. Furthermore, assorted food samples with varied macronutrient compositions were digested in order to evaluate the effect of reduced pancreatin and bile conditions on the digestibility of macronutrients (protein, starch, and lipid) and the subsequent release of minerals, in comparison with the standard INFOGEST 2.0 conditions. Ensuring an accurate assessment of Fe and Zn bioaccessibility is crucial for evaluating the nutritional potential of foods and providing critical information for public health, as these essential minerals support vital physiological functions and their deficiencies can lead to adverse health outcomes.

## 2 Material and methods

### 2.1 Samples

To evaluate the effects of different digestion conditions on macronutrient digestion and subsequent Fe and Zn release, food samples with diverse macronutrient compositions (*e.g.* protein, starch and lipids) were used. The selected samples were white long-grain rice, oats, potato, sweet potato, black turtle beans, pinto beans, Brazil nuts, cashew nuts, spinach, and kale. All products were purchased from local supermarkets in the UK.

#### 2.1.1 Cooking preparation

The samples were prepared to reflect their typical form of consumption, without the addition of other ingredients such as salt, oil, or seasonings that could introduce additional Fe and Zn. Bottled natural mineral water, also purchased from local supermarkets, was used throughout the cooking process. The cooking preparations were as follows:

• **Rice:** About 350 g of rice was rinsed and then simmered with 900 mL of water over a medium heat until all the water had been absorbed (approximately 20 min).
• **Oat porridge:** About 150 g of oats was simmered with 900 mL of water over low heat, stirring continuously for 3 min.
• **Potatoes:** The potatoes and sweet potatoes were peeled, cut, and then simmered in boiling water for 20 and 15 min, respectively. After cooking, the water was drained.
• **Beans:** About 350 g of black turtle beans and pinto beans were rinsed and then simmered with 1 L of water over high heat. After 70 min, an additional 1 L of water was added, and the heat was reduced. The beans continued cooking until the broth thickened, resulting in a volume ratio of approximately 50 % broth and 50 % grains.
• **Nuts:** No cooking was employed for Brazil nuts and cashew nuts, as they are typically consumed *in natura*.
• **Spinach and kale:** The spinach and kale were steamed until tender (approximately 5 and 12 min, respectively).

#### 2.1.2 Samples pre-treatment

The samples were minced in a food processor to simulate mastication, as indicated in the INFOGEST 2.0 protocol (Brodkorb et al., 2019). Aliquots of these samples were weighed into centrifuge tubes in which the *in vitro* digestion would be processed. Then, they were rapidly frozen with liquid nitrogen and stored at −20 °C until further assays. This procedure minimizes damage caused by slow freezing, which forms large ice crystals in the extracellular regions, affecting food microstructures and consequently could influence the bioaccessibility of nutrients (Li, Zhu, & Sun, 2018). A portion of each sample was freeze-dried until constant weight and then finely ground for total composition analysis. Samples were also weighed before and after drying to determine their moisture content.

### 2.2 Food composition analysis

The protein content in the samples was determined based on nitrogen quantification by the Dumas method using an elemental analyser (CN analyser, LECO). The nitrogen results were multiplied by a nitrogen conversion factor of 6.25 to estimate the protein content (Mariotti, Tomé, & Mirand, 2008).

The lipid content in the samples was determined by Soxhlet method using hexane solvent in a SOXTHERM extraction system (Gerhardt, Germany). Data on starch content were obtained from McCance and Widdowson's The Composition of Foods Integrated Dataset (CoFID) (Public Health England, 2021).

### 2.3 Removal of minerals from reagents

Muleya et al. (2021) had previously demonstrated the high contribution of pancreatin and bile salts to the concentrations of Fe and Zn in digestive fluids. Therefore, processing methods to remove minerals from pancreatin and bile were evaluated, aiming to reduce the mineral background in the fluid. This approach would enable the treated reagents to be used in the digestion while maintaining standard INFOGEST 2.0 conditions. Thus, sonication, centrifugation and Chelex® 100 resin treatments were evaluated as potential strategies to reduce the mineral concentration in these reagents.

Initial tests were conducted using pancreatin suspension at a concentration of 58.8 mg/mL, which corresponded to approximately 258 U/mL of trypsin activity.

At first, to evaluate the centrifugation and ion-exchange resin treatments, a 10 mL volume of pancreatin suspension was centrifuged at 4500 ×*g* for 10 min. Then, a 5 mL aliquot of the supernatant was mixed with 250 mg of Chelex® 100 resin and incubated in a shaking water bath at 18 °C for 1 h. The mixture was then centrifuged again at 4500 ×*g* for 10 min, and the supernatant was carefully separated from the solid resin using a pipette. Both the remaining solution that was centrifuged and the one treated with Chelex® 100 resin were reserved for Fe and Zn determination, as well as for trypsin activity analysis. A similar treatment was performed for bile, starting with a 5 mL solution of 40 mM bile salts mixed with 250 mg Chelex® 100 resin. After incubation, the mixture was decanted, and the solution was separated from the solid resin.

Subsequently, the sonication prior to centrifugation treatment of pancreatin solution was evaluated. A volume of 5 mL of pancreatin suspension was sonicated in an ultrasonic bath for 5 min and then centrifuged at 4500 ×*g* for 10 min. Another 5 mL of pancreatin suspension was centrifuged at 4500 ×*g* for 10 min without prior sonication. The solutions were then analysed to determine the Fe and Zn concentrations and trypsin activity.

Additionally, the optimal treatments were applied to a larger amount of pancreatin, typical of quantities required under standard INFOGEST 2.0 conditions (Brodkorb et al., 2019).

#### 2.3.1 Trypsin activity in pancreatin

To evaluate effects of the treatments on the pancreatin, trypsin activity was measured following the INFOGEST 2.0 recommendations. Briefly, 100 μL of each solution (centrifuged with and without prior sonication and treated with Chelex® 100 resin), after appropriate dilution, was mixed with 2.6 mL of Tris-HCl buffer (pH 8.1) and 300 μL of 10 mM TAME in a cuvette. The absorbance increase at 247 nm were recorded continuously for 10 min. The enzymatic activity was calculated based on the slope of the linear portion of the curve (Brodkorb et al., 2019). A fresh pancreatin solution was also prepared and analysed as a control.

### 2.4 *In vitro* digestion methods for Fe and Zn bioaccessibility

The ten food samples were digested using two digestion conditions; 1. low pancreatin and bile conditions based on the modified method by Muleya et al. (2021), 2. High pancreatin and bile conditions based on standard INFOGEST 2.0 conditions. In both cases, stable isotopes of Fe and Zn were applied to enable discrimination between reagent and sample Fe and Zn.

#### 2.4.1 *In vitro* digestion under reduced pancreatin and bile conditions

Briefly, in the method proposed by Muleya et al. (2021), simulated digestion fluids were prepared with electrolytes and CaCl_2_ as recommended in the INFOGEST protocol. The simulated salivary fluid (SSF) contained α-amylase from Bacillus *sp.* (75 U/mL in the oral phase), the simulated gastric fluid (SGF) contained pepsin from porcine gastric mucosa (2000 U/mL in the gastric phase), the simulated pancreatin fluid (SPF) contained pancreatin from porcine pancreas (100 U/mL of protease activity in the intestinal phase), and the simulated bile fluid (SBF) contained bovine bile (2 mM of bile salts in the intestinal phase). These reagents were all obtained from Sigma Aldrich (Dorset, UK). Stable isotopes ^57^Fe and ^70^Zn (c.95 % enrichment), purchased from Isoflex (USA), were added to each simulated fluid (SSF, SGF, SPF, and SBF) at levels 10 times their concentration in the respective solution. In this way, the added isotopes reached sufficiently high concentrations to allow their quantification and clear differentiation from the naturally occurring isotopes in the solution. The isotopically labelled fluids were incubated overnight in a shaking water bath at 20 °C to ensure isotopic equilibration.

In this method, a reduced amount of pancreatin and bile were employed. The amount of pancreatin added was based on achieving 100 U/mL protease activity (based on a protease activity of 200 U/mg in pancreatin) rather than 100 U/mL of trypsin activity, as recommended by the INFOGEST protocol. The trypsin activity of the pancreatin used in this study was 4.1 U/mg meaning under reduced pancreatin conditions, trypsin activity achieved was 2.0 U/mL compared to 100 U/mL under standard INFOGEST conditions.

The concentration of bile salts in the intestinal phase was reduced from 10 mM to 2 mM. All the other conditions and reagents amounts, were maintained according to the standard INFOGEST conditions.

Food samples (2.5 g each) were previously weighed and stored at −20 °C (Section 2.1.2). For the nut samples, due to low moisture content, 0.5 g was mixed with 2.0 mL of Milli-Q water to make a 20 % dry matter slurry. All samples were defrosted and brought to room temperature prior to *in vitro* digestions. In the oral phase, samples were mixed with 2.488 mL of SSF and 0.012 mL of CaCl_2_, and then incubated at 37 °C in a shaking water bath for 2 min. This was followed by the gastric phase, in which 5 mL of SGF was added, the pH was adjusted to 3.0, and the mixture was incubation for 2 h. Subsequently, in the intestinal phase, 5 mL of SPF and 5 mL of SBF was added, the pH was adjusted to 7.0, and the mixture was incubation again for 2 h. As soon as digestion was completed, the tubes were placed on ice for 15 min to stop enzymatic activity. Then, the samples were centrifuged at 4500 ×*g* for 15 min, and supernatants were collected for determination of soluble mineral fractions and the digestibility of macronutrients (protein, lipids, and starch).

Three additional runs of cashew and Brazil nut digestions were performed to obtain sufficient material for lipid hydrolysis analysis, in which whole digestion samples, including both solid and soluble fractions, were required.

#### 2.4.2 *In vitro* digestion under standard INFOGEST 2.0 conditions

Samples were also digested using stable isotope approach under standard INFOGEST 2.0 conditions. However, to reduce the artefact caused by increased Fe and Zn in the standard INFOGEST 2.0, the procedures described in Section 2.3 that provided the best results for reducing minerals in pancreatin and bile were applied prior to preparing the SPF and SBF digestive fluids. Thus, recommended amounts of pancreatin (100 U/mL of trypsin activity in the intestinal phase) and bile (10 mM of bile salts in the intestinal phase), were applied. The use of gastric lipase is also recommended in INFOGEST 2.0, however, due to limited commercial availability, this enzyme was not included. (Brodkorb et al., 2019). These fluids were isotopically labelled, as in the digestion method with reduced reagents, by adding stable isotopes ^57^Fe and ^70^Zn at levels 10 times the estimated concentration in respective solutions. Digestions were performed on food samples, following the same procedure described in Section 2.4.1.

#### 2.4.3 Determination of Fe and Zn bioaccessibility using the dialysis method

In 2.4.1, 2.4.2, standard INFOGEST 2.0 was compared with reduced pancreatin and bile conditions to evaluate impact on macronutrient digestibility and subsequent Fe and Zn solubility. The procedure deemed to be optimal was then applied to the samples, now including the dialysis step to determine soluble low molecular weight Fe and Zn as a measure of bioaccessibility. The dialysis tubing, was filled with 17.5 mL of 0.05 M 1,4-piperazinediethanesulfonic acid disodium salt (PIPES) buffer (pH 6.7) and tied at both ends using a double knot.

The digestion followed the previously described procedure. However, during gastric digestions, after 90 min of incubation, dialysis tubing was added to the tubes, except for the blanks. The samples were then incubated for an additional 30 min. Subsequently, the intestinal digestion was carried out, with dialysis tubing remaining in the tubes until the end of the digestion. Finally, after stopping enzymatic activity in an ice bath, the dialysis tubing was removed and its contents were collected for mineral analysis.

#### 2.4.4 Macronutrient hydrolysis

To evaluate the protein hydrolysis during *in vitro* digestion, the remaining proteins present in the supernatants were precipitated by mixing with cold 5 % trichloroacetic acid (TCA). After centrifugation, the primary amino groups released during proteolysis were determined by OPA assay (Sousa et al., 2023). In addition, analytical blanks (digestion fluids without sample) were also analysed in order to discount possible contributions of primary amino groups originating from the digestion enzymes.

Starch digestibility was assessed by quantifying reducing sugars produced. Aliquots of supernatant were mixed to an equal volume of 0.3 M Na_2_CO_3_ to stop the enzymatic reaction. Then, these mixtures were centrifuged and analysed by DNS assay to determine the concentration of reducing sugars produced from starch amylolysis (Wood et al., 2012).

For lipid hydrolysis analysis, the whole digesta, including solid and soluble fractions, were freeze-dried. Lipids were extracted by adding 2 mL of hexane per gram of dried sample (2:1 *v*/*w* ratio), vortexing for 30 s, and centrifuged at 2400 ×*g* at 4 °C for 20 min. The supernatant was transferred to a new flask, and the extraction was repeated twice. Then, the combined extracts were filtered through a 0.22 μm syringe filter and dried under nitrogen atmosphere to remove the solvent, leaving only the lipid fraction, which was subsequently weighted. The dried lipid extracts were re-dissolved in a known volume of hexane and used for a lipid class analysis by HPTLC to determine the free fatty acids (FFA), monoacylglycerols (MAG), diacylglycerols (DAG), and triacylglycerols (TAG) in the digesta.

In addition, this extraction procedure was also performed on non-digested samples, which were used as a reference to calculate extraction efficiencies. These non-digested samples were the ones that had been minced to simulate mastication before being used for *in vitro* digestion.

### 2.5 Mineral concentration analysis

To determine the total Fe and Zn concentrations in the food samples, enzymes and bile, about 0.2 g of dried and ground food samples and 0.1 g of α-amylase, pepsin, pancreatin, and bile were acid-digested in a microwave oven using an oxidizing mixture of 3 mL of HNO_3_, 2 mL of H_2_O_2_ and 3 mL of H_2_O. The treated pancreatin and bile suspensions (Section 2.3), as well as soluble (2.4.1, 2.4.2) and dialyzable (Section 2.4.3) fractions from the *in vitro* digestion, were also acid-digested under same conditions, using 2 mL of sample mixed with 2 mL of H_2_O_2_ and 3 mL of HNO_3_.

The digested samples were diluted and analysed by Inductively Coupled Plasma Mass Spectrometry (ICP-MS, model iCAP Q, Thermo-Fisher Scientific). A rhodium standard solution (5 μg/L) was employed as the internal standard. The isotopes monitored for quantification were ^56^Fe, ^66^Zn, and ^103^Rh. Results of total concentrations were converted based on the weight and volume used for the acid-digestion and expressed in mg/kg.

For quality control, standard reference materials from the National Institute of Standards and Technology (NIST) were also analysed. Fe and Zn recoveries for wheat flour (SRM 1567b) were 85 % and 90 %, respectively, while those for tomato leaf (SRM 1573a) were 73 % and 88 %.

The mineral concentrations determined in enzymes and bile were used to estimate the contribution from each reagent to the concentrations of Fe and Zn in the digestion mixtures. The estimation was based on the digestion input of 2.5 g of food samples, for which the total volumes of oral, gastric and intestinal digestion phases were 5 mL, 10 mL, and 20 mL, respectively. First, the weights of each reagent required per digestion (w_reagent_) were calculated according to Eq. (1), where A_required_ is the required enzyme activity or bile concentration defined by INFOGEST 2.0, v_digestion phase_ is the volume of the respective digestion phase, and A_specific_ is the specific enzyme activity or bile concentration. Subsequently, the concentrations of Fe and Zn in the digestion mixtures (C_digestion_) were calculated using Eq. (2), where w_reagent_ is the weight of each reagent, C_reagent_ is the previously determined mineral concentration in the respective reagent, and v_final_ is the final digestion volume.

(1) $$w_{\mathrm{reagent}}=\frac{A_{\mathrm{required}}\times v_{\mathrm{digestion phase}}}{A_{\mathrm{specific}}\;}$$

(2) $$C_{\mathrm{digestion}}=\frac{w_{\mathrm{reagent}}\times C_{\mathrm{reagent}}}{v_{\mathrm{final}}\;}$$

For soluble and dialyzable fractions after *in vitro* digestion, additional isotopes, ^57^Fe and ^70^Zn, were monitored and data were processed according to Muleya et al. (2021), which provides the detailed calculations. Calculations were based on the native (^56^Fe and ^66^Zn) and applied isotopes (^57^Fe and ^70^Zn). Iron and Zn soluble and dialyzable concentrations were then expressed as percentages of the total Fe and Zn concentrations in each food sample.

### 2.6 Statistical analyses

The food samples were digested in triplicate, and results were expressed as means ± standard deviation. Data were analysed using a two-way analysis of variance (ANOVA) in XLSTAT, version 2022.4.1 (Addinsoft, Paris, France). The factors included sample type and method, and their interaction. Where significant interactions were observed, simple main effects were examined by conducting pairwise *t*-tests to compare methods within each sample type. Significance was set at *p* < 0.05. For comparison of dialysable fractions across samples, one-way ANOVA followed by Tukey's post-hoc test (p < 0.05) was performed. Meanwhile, for the comparison of lipid class results within the same food matrix, a Student's t-test (p < 0.05) was performed.

## 3 Results and discussion

### 3.1 Food composition

Samples of diverse macronutrient composition, including cereals, tubers, legumes, nuts, and leafy greens, were selected to evaluate the effect of reduced pancreatin and bile concentrations on macronutrient digestibility and subsequent Fe and Zn solubility. The results of food composition, including starch content from McCance and Widdowson's The Composition of Foods Integrated Dataset (CoFID) (Public Health England, 2021), are presented in Table 1. The moisture content was used to calculate the protein, lipid, Fe and Zn concentrations on a wet weight basis, representing how the foods are consumed.

**Table 1:** Moisture, protein, lipids, Fe and Zn content in the cooked food samples based on wet weight.

Sample	Moisture %	Protein %	Lipids %	Starch %⁎	Fe (mg/kg)	Zn (mg/kg)
Rice	69.5 ± 0.2	2.212 ± 0.008	0.0293 ± 0.0002	31.1	1.1 ± 0.4	1.88 ± 0.03
Oat porridge	83.0 ± 0.4	2.12 ± 0.02	0.57 ± 0.01	70.4	6.77 ± 0.06	4.9 ± 0.2
Potato	82.0 ± 0.2	1.37 ± 0.02	0.00609 ± 0.00001	16.7	3.4 ± 0.1	2.42 ± 0.04
Sweet potato	81.19 ± 0.05	0.687 ± 0.004	0.078 ± 0.002	3.6	2.3 ± 0.2	1.50 ± 0.06
Black beans	78.4 ± 0.5	5.51 ± 0.05	0.300 ± 0.004	ND	14.9 ± 0.2	7.48 ± 0.01
Pinto beans	82.5 ± 0.1	3.79 ± 0.02	0.251 ± 0.004	ND	11.5 ± 0.8	6.1 ± 0.1
Brazil nuts	1.99 ± 0.02	16.13 ± 0.05	60.3 ± 0.2	0.7	27.9 ± 0.8	41.3 ± 0.7
Cashew nuts	3.4 ± 0.1	19.59 ± 0.08	43.7 ± 0.6	13.5	64 ± 4	55 ± 2
Spinach	90.1 ± 0.3	3.392 ± 0.008	0.59 ± 0.08	0.2⁎⁎	22.5 ± 0.6	9.1 ± 0.1
Kale	85.3 ± 0.2	2.7674 ± 0.0003	0.84 ± 0.08	0.1⁎⁎	18 ± 1	11.5 ± 0.4

*n* = 3.

ND = no data.

⁎ Data from McCance and Widdowson's The Composition of Foods Integrated Dataset (CoFID) (Public Health England, 2021).

⁎⁎ Boiled.

Protein content ranged from 0.687 % to 19.6 %, lipid content from 0.078 % to 60.3 %, and starch content from 0.1 % to 70.4 %. Mineral contents also ranged considerably among the samples, with Fe between 1.1 and 64 mg/kg and Zn between 1.5 and 55 mg/kg. These results demonstrate the diversity of macronutrients in the samples, which enables a rigorous comparison of the effect of macronutrient digestion on mineral solubility. Moreover, although starch values were obtained from the dataset, they serve the purpose of demonstrating the compositional variability.

### 3.2 Mineral concentration in reagents

Table 2 shows the concentrations of Fe and Zn determined in the enzymes and bile used to prepare the digestive fluids. The specific activity of each enzyme and the concentration of bile salts are also presented. These values enable the estimation of the contribution from each reagent to the concentrations of Fe and Zn in the digestion mixtures.

**Table 2:** Fe and Zn contents of reagents used to prepare the digestive fluids and estimated contributions of each reagent to the Fe and Zn concentration in the final digestion mixtures.

Parameter	α-amylase	Pepsin	Pancreatin	Bile	Treated pancreatin
Fe (mg/kg)	16.7 ± 0.3	22 ± 2	53.9 ± 0.9	12.7 ± 0.5	5781 μg/L
Zn (mg/kg)	12.41 ± 0.06	1.3 ± 0.1	181 ± 4	9.1 ± 0.5	26,647 μg/L
Specific activity (U/mg) or bile concentration (mmol/g)	701	2054	4.3	1.41	704 U/mL
**Digestion phase**	**Oral**	**Gastric**	**Intestinal**		
Enzyme activity (U/mL) or bile concentration (mM)	75	2000	100⁎	10	100⁎
Volume of digestion phase (mL)	5	10	20	20	20
Amount of enzyme (mg) or bile per digestion (mg)	0.53	9.74	465	142	2.84 mL
Final digestion volume (mL)	20	20	20	20	20
**Estimated reagent contributions**					
Fe (μg/L)	0.447	11	1253	90	821
Zn (μg/L)	0.332	0.63	4209	64	3784

**Treated pancreatin:** Pancreatin suspension (1 mg/mL) centrifuged with prior sonication.

⁎ Based on trypsin activity.

Muleya et al. (2021) obtained substantially higher concentrations of Fe (226 mg/kg) and Zn (75 mg/kg) in pepsin, as well as Fe (111 mg/kg) in bile, compared to the values obtained in this study. These variations can be attributed to the different batches of reagents used. Regarding the Fe and Zn contribution to the digestion mixtures, α-amylase and pepsin showed the least contributions, while the pancreatin remained the main source of minerals.

Considering the reductions in pancreatin and bile amounts proposed by Muleya et al. (2021) (Described in Section 2.3), the estimated contribution of Fe and Zn from pancreatin decreased by approximately 46-fold (Fe: 27 μg/L; Zn: 91 μg/L), and that from bile by 5-fold (Fe: 18 μg/L; Zn: 13 μg/L). These mineral decreases refer only to the reduction in reagent amounts, and the estimations are based on the total mineral content in the reagents as supplied, without any removal treatment applied.

### 3.3 Removal of minerals from reagents

Due to the intrinsically high mineral content of pancreatin and bile salts, using the higher reagent amounts recommended under standard INFOGEST 2.0 conditions would substantially increase the background levels of Fe and Zn, which interfere with bioaccessibility measurements. Therefore, strategies to reduce the intrinsic mineral content of these reagents were evaluated by subjecting pancreatin and bile salts to treatments prior to their use in the digestion.

#### 3.3.1 Pancreatin

The results of the fresh pancreatin (used as a control) and the two treatment experiments, which aimed to reduce the mineral content in pancreatin, are shown in Table 3.

**Table 3:** Mineral concentration and trypsin activity in pancreatin after treatments.

	Treatment	Fe (mg/kg)	Zn (mg/kg)	Trypsin activity (U/mg)
Control	Fresh pancreatin	53.9 ± 0.9	181 ± 4	4.3
Centrifugation and resin exchange	Centrifugation	21.0 ± 0.6	106 ± 2	3.9 ± 0.1
	Chelex® 100 resin	18 ± 1	41.08 ± 0.07	1.7 ± 0.2
Sonication prior to centrifugation	Centrifugation	22.2 ± 0.2	101 ± 3	2.9 ± 0.1
	Sonication and centrifugation	24.4 ± 0.1	120 ± 1	3.5 ± 0.1

**Centrifugation and resin exchange:** Pancreatin suspension (58.8 mg/mL) centrifuged at (4500 ×g, 10 min and centrifuged pancreatin treated with Chelex® 100 resin (5 mL:250 mg).

**Sonication prior to centrifugation:** Pancreatin suspension (58.8 mg/mL) centrifuged with or without prior sonication.

In the evaluation of centrifugation and ion-exchange resin treatments, the reductions of Fe and Zn in pancreatin by centrifugation, compared with the control, were 61 % and 41 %, respectively. In contrast, the reductions after Chelex® 100 resin treatment were 67 % and 77 %. However, although Chelex® 100 resin improved the mineral reduction, the trypsin activity decreased from 4.3 U/mg in the fresh pancreatin to 1.7 U/mg after treatment, corresponding to a 60 % reduction, making it unsuitable for digestion. On the other hand, the trypsin activity after centrifugation was only 9 % lower than that of fresh pancreatin.

The effect of the sonication prior to centrifugation treatment was evaluated (Table 3). Mineral concentrations in pancreatin subjected to sonication prior to centrifugation were 10–18 % higher than in pancreatin centrifuged without prior sonication. However, this treatment increased the trypsin activity by 21 % (from 2.9 to 3.5 U/mg). Although mineral removal was slightly lower with sonication, the higher trypsin activity allows for the use of a smaller amount of pancreatin to achieve the required digestion conditions, consequently reducing its mineral contribution to the digesta.

Therefore, sonication followed by centrifugation was chosen to remove minerals from pancreatin while minimizing trypsin activity losses. Sonication and centrifugation of pancreatin is also recommended in a recently established analytical workflow based on the INFOGEST method to determine protein digestibility (Sousa et al., 2023).

To employ the combined sonication and centrifugation strategy for the digestions, larger amounts of treated pancreatin should be produced. For this purpose, the pancreatin-to-water ratio was increased to obtain a suspension with suitable enzymatic activity for the preparation of digestive fluids under the standard INFOGEST 2.0 conditions. Thus, a batch prepared at a ratio of 4.375 g of pancreatin to 25 mL of water (175 mg/mL) was also tested. The trypsin activity in the supernatant of sonicated and centrifuged pancreatin was 704 U/mL, corresponding to 4.02 U/mg. The Fe and Zn concentrations were 33.03 mg/kg and 152.27 mg/kg respectively. The variation in the results compared to the 2nd experiment is possibly due to differences in the mass and volume used in the treatment, since the change in the solid-to-liquid ratio could affect the equilibrium of mineral solubilization. Lower pancreatin concentrations yielded larger reductions in mineral contents, whereas higher concentrations resulted in less pronounced reductions. However, the higher concentration of pancreatin is required to achieve the trypsin activity recommended in the standard INFOGEST 2.0 protocol.

Table 2 also shows the estimated contribution of the treated pancreatin suspension to the concentrations of Fe and Zn in the digestion mixtures under the standard INFOGEST 2.0 conditions. These values were 34 % and 10 % lower than estimated contribution from untreated pancreatin under the same conditions.

In terms of bile, the Fe and Zn concentrations in bile treated with Chelex® 100 resin were 14 ± 2 mg/kg and 8 ± 1 mg/kg, respectively, showing no significant difference compared with 12.7 ± 0.5 mg/kg for Fe and 9.1 ± 0.5 mg/kg for Zn in the fresh bile (Table 2). Therefore, the Chelex® 100 resin was not effective in removing minerals from bile. However, as previously mentioned, the total concentration of these elements in the bile batch used in this study were significantly lower than that reported by Muleya et al. (2021). Moreover, the results in Table 2 indicate that this reagent could be used without a high Fe and Zn contribution to the digestive fluids, especially in comparison to the pancreatin.

### 3.4 Comparison of *in vitro* digestion methods: Reduced pancreatin and bile conditions *vs.* standard INFOGEST conditions

#### 3.4.1 Macronutrient digestibility

**Fig. 1:**
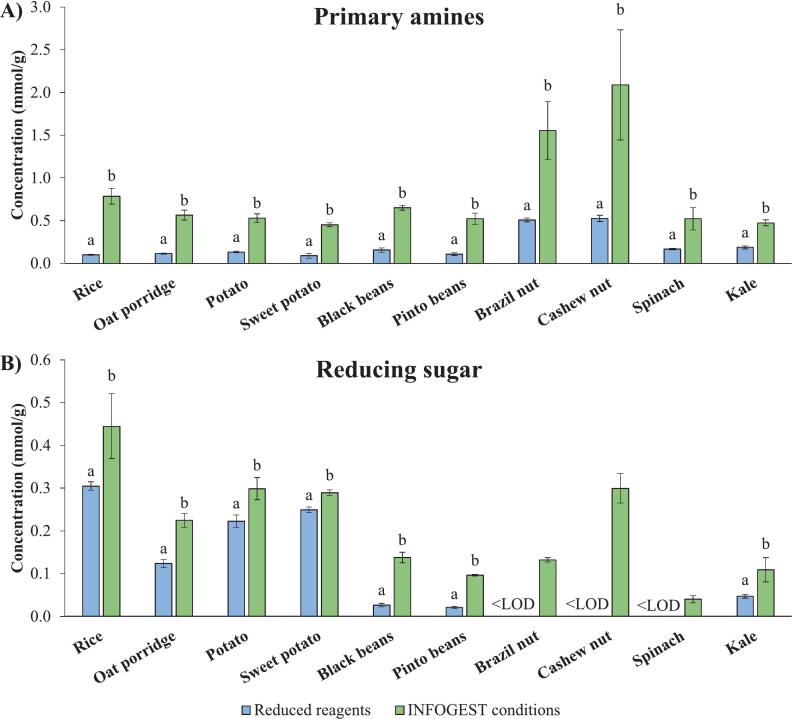
Primary amines (A) and reducing sugars (B) in food samples digested using the reduced pancreatin and bile and the standard INFOGEST 2.0 conditions (wet weight basis).^a,b^ Different superscripts within the same sample indicate significant differences (*p* < 0.05). LOD for reducing sugar: 0.04 mmol/g (nuts); 0.008 mmol/g (spinach).

The results of protein and starch digestibility are presented in Fig. 1A and B, respectively. The concentrations of primary amines and reducing sugars were statistically greater in all food samples digested using the method with treated pancreatin under standard INFOGEST 2.0 conditions (Fig. 1) compared to reduced enzyme conditions. This demonstrates that reducing the amounts of pancreatin and bile in the fluids can affect protein and starch hydrolyses. However, the two-way ANOVA showed significant effects of sample type and method on the digestibility of both macronutrients. In addition, a significant interaction between sample type and method was observed. Thus, although a general trend of increased digestibility was observed using the method under standard INFOGEST 2.0 conditions, the extent of the increase in digestibility was also influenced by the food type.

In general, protein digestibility was affected more than starch digestibility. The concentrations of primary amines increased 2 to 7-fold under INFOGEST 2.0 conditions. In contrast, reducing sugar increased up to 2-fold under INFOGEST 2.0 conditions, except in bean samples, where it increased approximately 5-fold.

Furthermore, cashew and Brazil nut samples were selected to evaluate the effect of the digestion method modification on lipid digestibility due to their high fat content. In this case, the whole digesta samples were analysed instead of only the soluble fraction. The extraction efficiencies, based on the values obtained from non-digested samples also submitted to the hexane extraction, ranged from 45 % to 68 % for Brazil nuts. These values were lower than expected, indicating that further investigation is needed. This reduced efficiency was probably influenced by intact cell walls or higher lipid content in this sample. Although extraction efficiencies obtained for Brazil nuts were low, those for cashew nuts were satisfactory, ranging from 81 % to 112 %, thereby allowing the evaluation of lipid digestibility under the different *in vitro* digestion conditions.

**Fig. 2:**
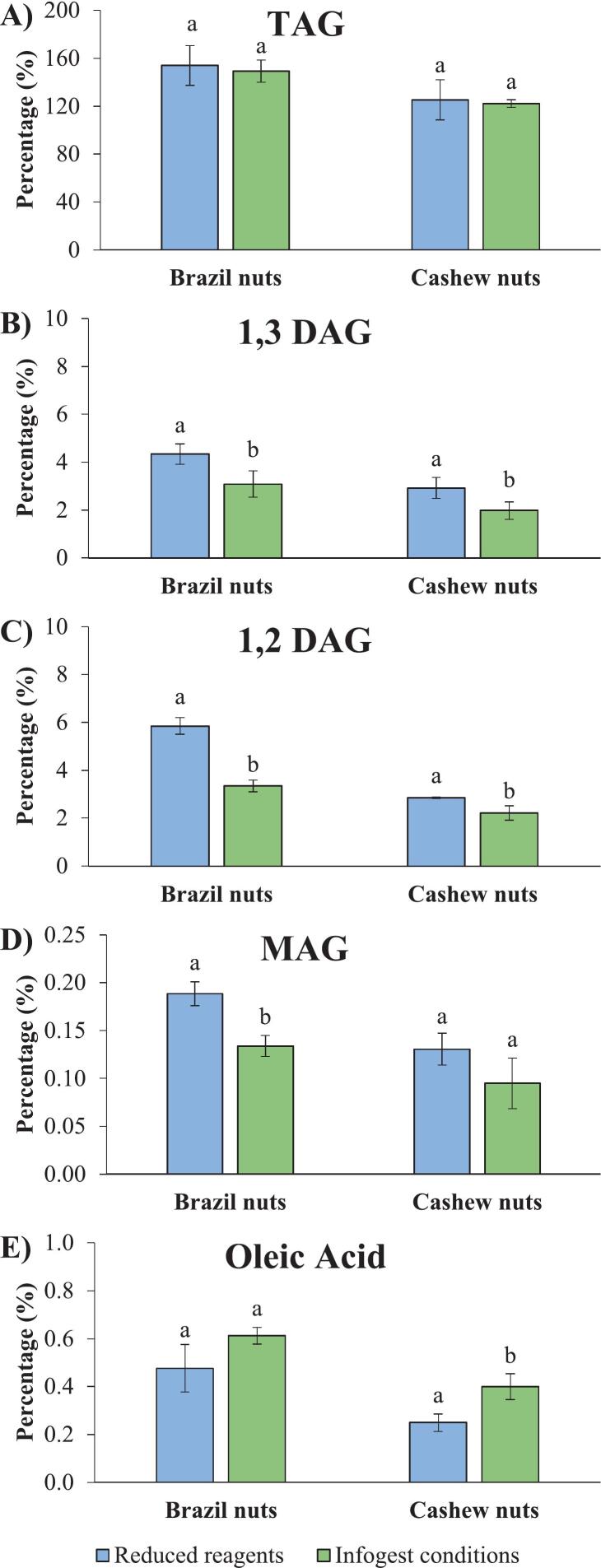
Lipid class analysis by HPTLC of nut samples digested using the reduced pancreatin and bile and the standard INFOGEST 2.0 conditions. A) Triacylglycerols (TAG), B) 1,3-Diolein (1,3 DAG), C) 1,2-Dioleoyl-rac-glycerol (1,2 DAG), D) Monoolein (MAG), and E) Oleic acid (FFA). (Wet weight basis).^a,b^ Different superscripts indicate significant differences (p < 0.05).

Lipid class analysis by HPTLC was performed to determine free fatty acids (FFA), monoacylglycerols (MAG), diacylglycerols (DAG), and triacylglycerols (TAG) in the samples digested using both methods evaluated in this study (Fig. 2). The results were expressed as relative percentages of the total extracted lipid mass. This approach allowed the comparison of the lipid class distribution profiles independently of extraction efficiency, which may have affected the absolute concentrations.

In lipid digestion, TAGs are gradually hydrolysed, with each step releasing one FFA, first forming DAG, then MAG, and finally producing glycerol and a last FFA (Grundy et al., 2016; Zheng et al., 2023). No significant reductions in TAG levels were observed for both methods, and only low percentages of lipid hydrolysis products (FFA, MAG, and DAG) were obtained, ranging from 0.09 % to 5.9 %. These results indicate low lipid digestibility in these samples, despite differences in pancreatin and bile conditions.

**Fig. 3:**
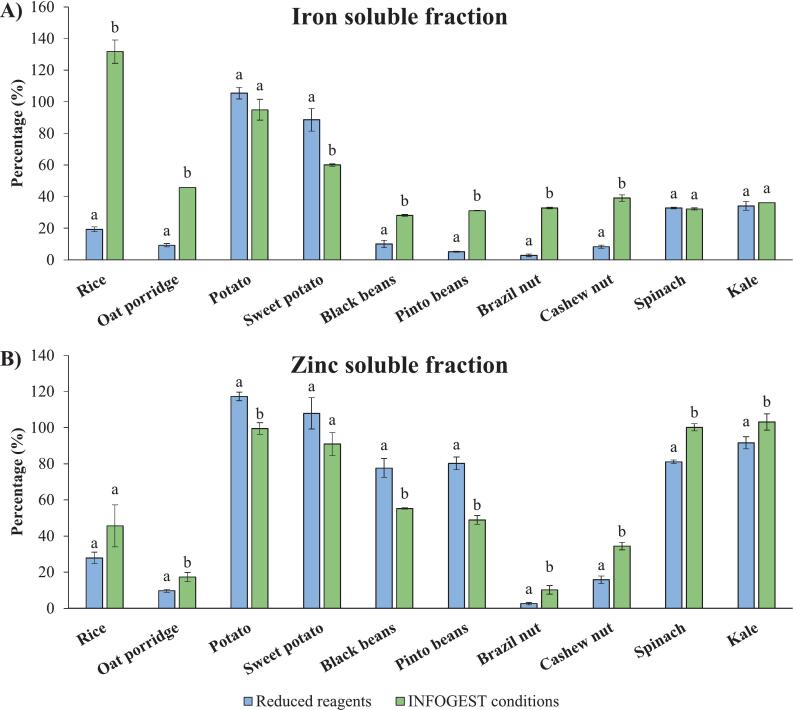
Fe (A) and Zn (B) soluble fractions in food samples digested using the reduced pancreatin and bile and the standard INFOGEST 2.0 conditions. ^a,b^ Different superscripts within the same sample indicate significant differences (p < 0.05).

Low lipid digestibility could be expected for the samples digested under reduced pancreatin and bile concentrations. However, even lower concentrations of DAG (Figs. 2B and 3C) and MAG (Fig. 2D) were observed in samples digested under INFOGEST 2.0 conditions. The decrease in intermediate products levels may suggest reduced digestibility under higher amounts of pancreatin and bile, which seems contradictory, since pancreatin and bile provide lipase enzymes and bile which should promote lipid emulsification and hydrolysis. It is possible that some lipase activity may have been lost during the centrifugation of the pancreatin suspension used for the digestions under the standard INFOGEST conditions, which could have reduced lipid hydrolysis. In addition, the absence of gastric lipase could also affect the lipid hydrolysis in both conditions. Gastric lipolysis contributes to the digestion of TAGs and facilitates the subsequent action of pancreatic lipase on lipid substrates (Brodkorb et al., 2019).

On the other hand, no significant difference was observed for the percentage of oleic acid (FFA) in Brazil nut samples digested under both methods, while higher levels of oleic acid were found in cashew nut samples digested using standard INFOGEST 2.0 conditions. This finding could suggest a more complete lipolysis of cashew nuts under higher pancreatin and bile conditions, resulting in reduced accumulation of intermediates (DAG and MAG) and increased formation of end-products (FFA).

Overall, the digestion method using reduced amounts of reagents altered the extent of macronutrient hydrolysis. Consequently, the assessment of Fe and Zn bioaccessibility may also be affected, since these minerals can be associated with macronutrients in the food matrix, and their hydrolysis products play a crucial role in mineral solubilization and bioaccessibility. These findings are relevant to the subsequent evaluation of mineral solubility.

Moreover, considering that the reduction in pancreatin and bile affect the hydrolysis of each macronutrient to a different extent, it can be asserted that there is a threshold of enzyme activity beyond which any increase does not yield significant change. For example, Ariëns et al. (2021) demonstrated that a 10-fold reduction in trypsin activity, from 100 U/mL to 10 U/mL, did not significantly affect protein digestibility. In the present study, a 46-fold reduction in trypsin activity was applied and this perhaps was too low to achieve comparable macronutrient hydrolysis with the standard INFOGEST 2.0 conditions. However, findings from Ariens demonstrate that reasonable reductions in pancreatin might still be permissible. Therefore, further studies could be conducted to evaluate the possibility of reducing pancreatin to levels that do not affect overall macronutrient breakdown.

#### 3.4.2 Fe and Zn released after *in vitro* digestion

For soluble Fe (Fig. 3A), two-way ANOVA also revealed highly significant effects of sample type and method used as well as a significant interaction between sample type and method (*p* < 0.0001). These findings indicate that soluble Fe differed substantially across sample types and that the pancreatin and bile conditions strongly influenced outcomes. Importantly, the significant interaction demonstrates that the effect of method was not consistent across samples. For example, soluble Fe was not changed for spinach, kale and potato while it increased under standard INFOGEST 2.0 conditions for rice, oat porridge, black beans, pinto beans, Brazil nuts and cashew nuts and decreased for sweet potato. This suggests that both the type of food matrix and the pancreatin and bile conditions contribute to variability in the proportion of soluble Fe.

For soluble Zn (Fig. 3B), the type of food sample was again a highly significant factor (p < 0.0001), confirming marked differences across samples. In contrast, there was no overall main effect of pancreatin and bile conditions (*p* = 0.587), indicating that, on average, the two methods did not differ in their proportion of soluble Zn for each sample type. However, there was significant interaction (p < 0.0001) between sample type and method which shows that the influence of method varied to some extent depending on the sample type, with certain samples showing differences between methods despite the absence of an overall method effect.

Interestingly, when observing the pattern of Fe and Zn solubility across the samples (Fig. 3A and B), those within the same food group (cereals, tubers, legumes, nuts, and leafy greens) tended to show similar responses, suggesting that mineral release is strongly influenced by the type of food matrix.

In the supplementary material (Fig. S1), the recovery of the applied stable isotopes in different food matrices after *in vitro* digestion under both reduced-reagent conditions and standard INFOGEST conditions is presented. The ^57^Fe and ^70^Zn recoveries were not consistent across food samples, and variations in the direction of recovery were observed. These variations indicate that the ligand environment is highly sensitive to changes in the mineral background and to binding compounds introduced by the higher reagent levels, as well as to the breakdown products released during food digestion.

There are many intrinsic characteristics of food samples that may affect the mineral release. Mineral bioaccessibility and macronutrient digestibility can be influenced by particle size of samples submitted to digestion process (Cilla et al., 2019). Moreover, the hydrolysis products of macronutrients can interact with minerals to enhance their solubility (Zhang, Stockmann, Ng, & Ajlouni, 2023). On the other hand, anti-nutritional compounds (*e.g.* dietary fibre, polyphenols, and phytate) may bind to these elements, forming insoluble complexes (Rousseau, Pallares Pallares, Vancoillie, Hendrickx, & Grauwet, 2020; Zhang et al., 2021). Additionally, the localization of minerals within the food matrix and their distribution across cellular compartments can also impact their solubility (Holland et al., 2020; Shen et al., 2023).

**Fig. 4:**
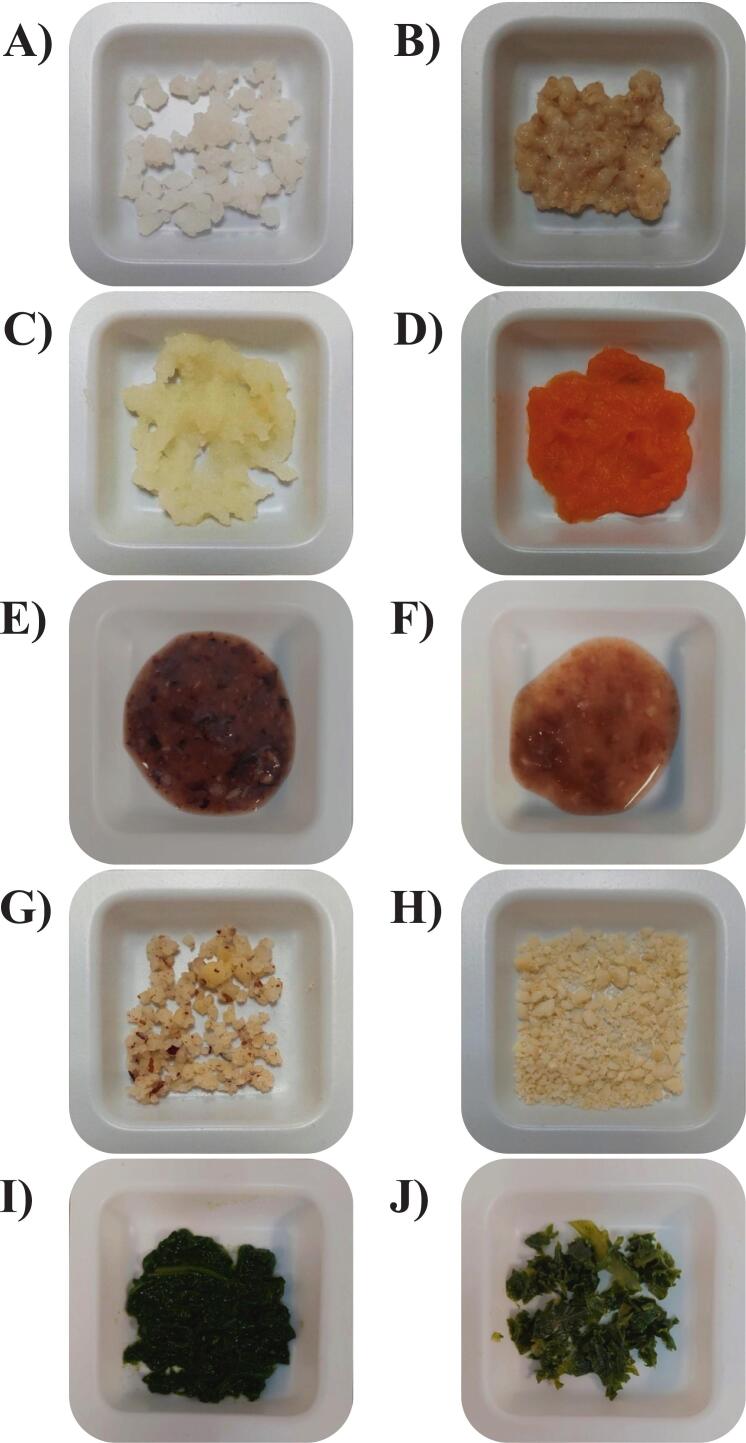
The minced food samples used for *in vitro* digestion. (A) white long-grain rice, (B) oats porridge, (C) potato, (D) sweet potato, (E) black turtle beans, (F) pinto beans, (G) Brazil nuts, (H) cashew nuts, (I) spinach, and (J) kale.

In cereals, such as rice and oat, minerals are more concentrated in the outermost layer, such as the aleurone and embryo. However, minerals could be more abundant overall in the endosperm, the inner part of the grain, due to its larger volume (Deng, Nagy, & Yu, 2023; Saccomanno et al., 2017; Shen et al., 2023). In order to mimic mastication, the samples were minced in a food processor, as indicated in the INFOGEST 2.0 protocol (Brodkorb et al., 2019). The minced food samples are shown in Fig. 4. Even after mincing, small intact grain particles were still visible in the rice and oat porridge samples (Fig. 4A and B). Therefore, the digestion using larger amounts of pancreatin and bile (Fig. 3) could have facilitated better the release of minerals physically trapped in the grain endosperm, increasing the soluble fraction compared with the low enzyme conditions.

In addition, cereals contain anti-nutritional compounds. In particular, phytic acid content has demonstrated a significant negative correlation with Fe and Zn bioaccessibility (Hemalatha, Platel, & Srinivasan, 2007; Nguyen & Watts-Williams, 2025). Moreover, mineral bioaccessibility is influenced by the molar ratio of phytate to Zn and/or Fe. In general, higher phytate-to-mineral ratios are associated with lower bioaccessible fractions (Nguyen & Watts-Williams, 2025). Low soluble mineral fractions were observed in rice and oat porridge, except for Fe in rice digested under INFOGEST 2.0 conditions, which was completely soluble. This reflects differences in the food matrix. The structure of rice facilitates Fe release during digestion, while the higher content of anti-nutritional compounds in oats may limit mineral solubility.

The potatoes, in contrast to rice and oat porridge, formed a more homogeneous paste during the mincing procedure (Fig. 4C and D). Thus, they may have had the mineral release facilitated by the higher surface area, even at the lowest reagent concentrations, resulting in high Fe and Zn soluble fractions (Fig. 3). Studies have also demonstrated that peptides released from potatoes protein digestion can bind to minerals offering greater stability against precipitation (Li et al., 2025). However, decreases in Fe and Zn solubility were observed under INFOGEST 2.0 conditions, even with a greater protein digestibility. This suggests that other components released from the food matrix under higher reagent conditions could inhibit the positive effects of protein hydrolysis products. For instance, potato tubers contain phenolic compounds, which may form insoluble complexes with minerals, potentially reducing their solubility during digestion (Andre et al., 2015).

Another hypothesis is that the higher concentration of chelating compounds introduced by the increased amounts of pancreatin and bile could have decreased Fe and Zn solubility. Muleya et al. (2021) have previously demonstrated the mineral binding effect of reagents used in digestion fluids. However, this effect would be expected across all samples subjected to the same reagent concentrations. Possibly, the lower protein content of potatoes tubers (Table 1) may have limited the formation of peptide-mineral complexes, which help maintain mineral solubility even in the presence of additional chelating agents. It is important to highlight that the digestion environment is a complex system involving dynamic equilibria and competition among various compounds for mineral binding, which can influence solubility outcomes in a matrix dependent manner.

Legumes contain proteins and anti-nutritional compounds, such as dietary fibres, polyphenols and phytic acid, that can affect the mineral bioaccessibility (Rousseau et al., 2020). While some legume peptic hydrolysates may effectively solubilise Fe and Zn, the presence of anti-nutritional compounds may bind to these elements and form insoluble complexes (Zhang et al., 2021). Rousseau et al. (2020) showed that phytic acid was mainly responsible for the decrease in Fe and Zn solubility. Thus, the low soluble Fe fractions in beans observed in Fig. 3A could be attributed to this compound. Notably, Zn soluble fractions (Fig. 3B) were higher than those of Fe, implying differences in mineral interactions.

Under INFOGEST 2.0 conditions, while the soluble Fe fractions in beans increased, the Zn solubility decreased (Fig. 3). This finding suggests that the greater hydrolysed product concentrations, such as amino acids and peptides, could have preferentially promoted Fe solubility. On the other hand, even after treatment, pancreatin remained a significant source of minerals (Table 3), which could disturb the equilibrium in the solutions. The Zn concentration was more than 4 times higher than that of Fe. Thus, the presence of high concentrations of Zn from the reagents could shift complexation and precipitation equilibria because of competition for the available ligands responsible for maintaining the solubility of Zn released from the samples.

Regarding the leafy greens, the mincing procedure used to simulate mastication left parts of spinach and kale tissues relatively intact, as shown in Fig. 4I and J. Fe and Zn in these plants are stored in subcellular compartments (Yruela, 2013). Since plant cell walls are composed of fibres that are not degraded during digestion, some Fe and Zn could be physically trapped within the cells (Holland et al., 2020).

In contrast to the other vegetal samples, which also contain fibres, no significant differences were observed in the soluble Fe fractions of leafy greens samples digested using either method. These findings, along with the low soluble Fe fractions, suggest that Fe could be strongly bound to components of the vegetable matrix that resist enzymatic digestion, such as dietary fibres (Holland et al., 2020; Rousseau et al., 2020). Moreover, spinach and kale contain other anti-nutritional factors like phytic acid, oxalic acid, and tannin (Naseem et al., 2023; Subedi, Raychaudhuri, Fan, Ogedengbe, & Obanda, 2024).

On the other hand, even under reduced reagent conditions, Zn in leafy green vegetables exhibited higher solubility than Fe. The method using pancreatin and bile at INFOGEST 2.0 recommended levels led to a slight additional increase in its solubility, possibly due to its being less tightly bound to the plant matrix and more susceptible to interactions with hydrolysis products that enhanced its release into solution. In addition, antinutrient compounds such as phytic acid and pectin show higher affinity for Fe than for Zn, especially when Fe is present as a trivalent cation, which exhibits an even greater binding affinity. However, mineral abundance and ratios within the chelating matrix also play a role in determining these interactions. (Rousseau et al., 2020).

In nuts, the Fe and Zn solubility notably increased during digestion with higher reagents amounts (Fig. 3). The mincing procedure used to simulate mastication produced a coarsely ground sample with some intact fragments (Fig. 4G and H). Therefore, as observed for rice and oats, Fe and Zn could be physically trapped in the inner parts of the solid particles. These nuts are mainly composed of lipids and proteins, as shown in Table 1. Xavier, Furtado, Assunção, and Nascimento (2019) demonstrated that significant fractions of Fe and Zn in cashew nuts are associated with albumins, globulins, and glutelins. Moreover, one third of the Fe and Zn bound to these proteins in cashew nut were considered bioaccessible after *in vitro* gastrointestinal digestion. Therefore, the increase in protein digestibility observed in Fig. 1A could facilitate the release of these minerals associated with this macronutrient, enhancing their solubility. As Brazil nuts have a similar matrix and composition, Fe and Zn can also be associated with proteins. Therefore, as observed for cashew nuts, the increase in Fe and Zn solubility under standard INFOGEST conditions could be attributed to the enhanced digestibility of this macronutrient.

In addition, although lipid digestibility may vary, mineral nutrients are not lipophilic. Thus, their association with the fat fraction during *in vitro* digestion is not expected (Moreda-Piñeiro, Herbello-Hermelo, Domínguez-González, Bermejo-Barrera, & Moreda-Piñeiro, 2016). Therefore, as lipids are the main component of nuts (Table 1), the effect of lipid digestibility on Fe and Zn solubility is also likely physical, facilitating the release of these elements trapped in intact sample fragments.

#### 3.4.3 Dialysed fractions

The digestibility of macronutrients was improved under INFOGEST 2.0 conditions. However, the soluble Fe and Zn fractions in the digested samples depended on the food matrix, although a general tendency toward increased solubility was observed with the use of higher amounts of reagents.

**Fig. 5:**
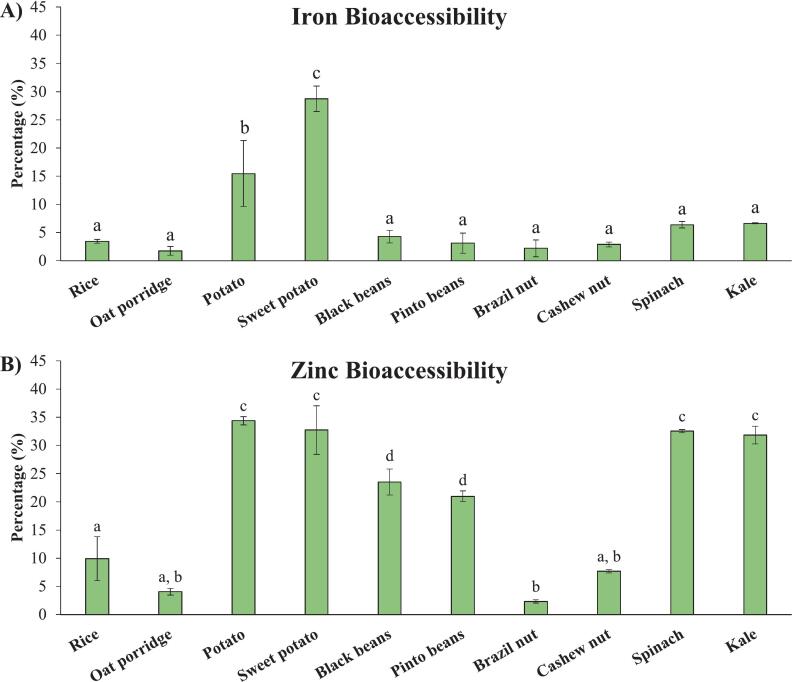
Dialysed Fe (A) and Zn (B) in food samples digested using the method with treated pancreatin under the INFOGEST 2.0 conditions (wet weight basis). ^a,b,c,d^ Different superscripts indicate significant differences (p < 0.05).

Although the analyses of the soluble fractions provided valuable insights into the macronutrient digestibility and mineral release, a dialysis step is recommended for the assessment of nutrient bioaccessibility, as it removes macromolecular-bound compounds that may not be absorbed by the organism (Brodkorb et al., 2019). Therefore, digestion using treated pancreatin under the INFOGEST 2.0 conditions, which was found to be more suitable for most of food matrices in assessing the bioaccessibility of these mineral nutrients, was selected for a second round of digestion, incorporating the dialysis step. Fig. 5A and B present the results for the dialysed fractions of Fe and Zn, respectively.

Potato and sweet potato presented the highest percentages of bioaccessible Fe, about 13 % and 19 %, respectively. The Fe bioaccessibility in other samples ranged from only 1.8 % to 6.7 %. Considering the absolute values, the percentages found in potatoes samples correspond to only 0.5 and 0.67 mg/kg (wet basis). In contrast, spinach and kale, despite having lower bioaccessible percentages (5.4 % and 5.6 %), contained greater bioaccessible Fe, of 1.4 and 1.18 mg/kg (wet basis), respectively.

Overall, the bioaccessibility of Zn was greater compared to Fe. The percentages of bioaccessible Zn in potatoes, beans and leafy green vegetables ranged from 21 % to 34.4 %, whereas lower fractions were observed in rice, oat and nuts, ranging between 2.3 % and 10 %. Similar to the results obtained for Fe, although potatoes and beans exhibited higher percentages of bioaccessible Zn, these values did not correspond to high absolute concentrations (0.49 to 1.8 mg/kg). In contrast, leafy vegetable not only showed high Zn bioaccessibility (32 % and 32.5 %, respectively) but also presented higher absolute concentrations in dialysate fraction (2.95 and 3.6 mg/kg).

## 4 Conclusion

The treatment based on sonication and centrifugation was effective in reducing minerals from pancreatin. However, *in vitro* digestion employing treated pancreatin under INFOGEST 2.0 conditions still presented a high mineral background, with estimated contribution of Fe and Zn from pancreatin being 30- and 41-fold higher than under reduced enzymes condition, respectively. Therefore, reagent mineral interactions remain during digestion, making the use of stable isotope approach necessary in both situations.

The processing methods tested to remove minerals from bile did not yield significant results. However, the bile batch used in this study presented lower Fe and Zn contents than values previously reported. This bile contributed to less than 7 % of the total mineral content in the digestion fluids, thus mineral removal treatments were not strictly necessary for bile. Therefore, mineral analysis is recommended prior to use of a new reagent batch in the digestion experiment, due to the high variability in mineral content.

The digestion of food samples with varied macronutrient compositions enabled the evaluation of the effects of using reduced amounts of pancreatin and bile. The digestibility of protein, starch, and lipid was improved under INFOGEST 2.0 conditions. However, Fe and Zn solubility were not consistently modified across matrices and across the two methods used, although, there was a general tendency toward increased solubility in the INFOGEST 2.0 method.

Interestingly, the mineral solubility behaviour varied depending on the sample matrix. This variability can be attributed to several factors, including the physical entrapment of Fe and Zn within solid particles or subcellular compartments, the chelation of minerals by hydrolysed products that prevent their precipitation, and the binding of minerals by antinutritional compounds or components of the digestion fluids, which reduces their solubility.

The digesta medium is a complex system, thus, the effects of enzyme activity cannot be fully isolated from those of background minerals, which compete for mineral binding in the system. The higher enzyme activity conditions employing treated pancreatin were chosen to ensure mineral release.

In conclusion, the use of standard INFOGEST 2.0 parameters is recommended to fully simulate enzymatic conditions, allowing optimal macronutrient breakdown. However, treatments identified in this study, specifically sonication and centrifugation of pancreatin, should be performed to reduce background minerals levels. Moreover, further studies could explore the possibility of reducing pancreatin to levels that do not compromise macronutrient breakdown. Thus, standardisation and optimization of the INFOGEST 2.0 method in terms of mineral bioaccessibility assessment is urgently needed to improve comparability of results across studies. Ensuring accurate assessment of mineral bioaccessibility is of critical importance, as misleading results may have serious implications for public health.

## CRediT authorship contribution statement

**Alexandre Minami Fioroto:** Writing – original draft, Methodology, Investigation, Formal analysis. **Molly Muleya:** Writing – review & editing, Supervision, Resources, Project administration, Methodology, Funding acquisition, Formal analysis, Conceptualization. **Lolita Wilson:** Writing – review & editing, Methodology, Investigation. **Kaja Kristensen:** Writing – review & editing, Methodology, Investigation. **Ruth Price:** Writing – review & editing, Methodology, Investigation. **David A. Gray:** Writing – review & editing, Resources, Methodology. **Eduardo Purgatto:** Writing – review & editing, Supervision, Funding acquisition. **Elizabeth H. Bailey:** Writing – review & editing, Supervision, Resources, Project administration, Methodology, Funding acquisition, Formal analysis, Conceptualization.

## Declaration of competing interest

The authors declare that they have no known competing financial interests or personal relationships that could have appeared to influence the work reported in this paper.

## Data Availability

Data will be made available on request.

## Appendix A Supplementary data

Supplementary material Image 1
