# Supplementary material for: Impact of reduced pancreatin and bile on Fe and Zn bioaccessibility assessment using the INFOGEST in vitro digestion method with stable isotopic labelling
Source: Food Chem X. 2026 Jan 2;33:103483. doi: 10.1016/j.fochx.2026.103483 (PMC12814079; doi:10.1016/j.fochx.2026.103483)
Supplement: Supplementary file 1 — Supplementary material [file mmc1.docx]

**Supplementary material**

The analytical blanks and samples were analysed by ICP-MS, and the applied isotopes (^57^Fe and ^70^Zn) were quantified according to Muleya et al. (2021). To evaluate changes in the ligand environment under the reduced-reagent conditions and the standard INFOGEST conditions across the different food matrices, the recovery of the applied stable isotopes (^57^Fe and ^70^Zn) was calculated based on their concentrations in the blanks and samples, as follows:

$$Recovery =\frac{applied isotopes in the blank}{applied isotopes in the sample}\times100\%$$

The recoveries of the applied stable isotopes (^57^Fe and ^70^Zn) in the different food matrices after *in vitro* digestion under reduced-reagent conditions and standard INFOGEST conditions are shown in Figure S1.

**Figure S1.** Recovered ^57^Fe and ^70^Zn in different types of food matrices after *in vitro* digestion under low (reduced reagents) and high pancreatin/bile conditions (standard INFOGEST).
